# Exact noise influenced soliton solutions of a high-order stochastic nonlinear Schrödinger equation with weak nonlocal nonlinearity in a non-Kerr medium

**DOI:** 10.1038/s41598-026-51492-0

**Published:** 2026-06-10

**Authors:** Mohammed H. Ali, Hamdy M. Ahmed, Soliman Alkhatib, M. Elsaid Ramadan, Islam Samir, Karim K. Ahmed

**Affiliations:** 1 Department of Basic Science, Higher Institute of Computer Science and Information Systems, Fifth Settlement, Cairo, Egypt; 2 Department of Mathematics and Engineering Physics, Higher Institute of Engineering, El-Shorouk Academy, El-Shorouk city, Egypt; 3 College of Engineering and Technology (CET), American University in the Emirates (AUE), Dubai intel Academic City, 503000 Dubai, UAE; 4 Department of Mathematics, Faculty of Science, Islamic University of Madinah, Medina, Saudi Arabia; 5 Department of Physics and Engineering Mathematics, Faculty of Engineering, Ain Shams University, Cairo, Egypt; 6 Department of Mathematics, Faculty of Engineering, German International University (GIU), New Administrative Capital, Cairo, Egypt

**Keywords:** Stochastic solitons, SNLSE, Optical fiber, Weak non-local nonlinearity, Modified extended mapping approach, Mathematics and computing, Optics and photonics, Physics

## Abstract

In this study, a high-order stochastic nonlinear Schrödinger equation (SNLSE) with weak non-local nonlinearity in a non-Kerr law medium is investigated. This model describes the propagation of solitons in nonlinear optical fibers under stochastic effects and higher-order nonlinear interactions. To obtain analytical solutions, a wave transformation together with symbolic computations and the modified extended mapping method (MEMM) is employed. As a result, various exact wave solutions are derived, including bright, dark, singular, periodic, and rational-type solitons. A rigorous linear stability analysis is performed using perturbation theory and dispersion relation analysis, demonstrating that the obtained solutions are linearly stable under small perturbations. The graphical behavior of the solutions under different parameter settings is also presented to illustrate the dynamical characteristics of the model. The results confirm the efficiency and reliability of the proposed method in handling high-order stochastic nonlinear Schrödinger-type equations.

## Introduction

In several scientific fields, Nonlinear partial differential equations (NPDEs) are used to simulate a variety of scientific processes, including quantum mechanics, chemical physics, mathematical physics, and optical fibers^1–5^. The importance of a recent study on “Soliton Solutions of NPDEs” has been recently highlighted. Soliton solutions to NPDEs can now be retrieved systematically thanks to the usage of symbolic computing programs. Research on wave propagation using optical fibers is essential to comprehending and controlling communication networks. Recently, various integration techniques have been presented to handle NLPDEs such as the extended direct algebraic method^6^, the Sardar sub-equation method^7,8^, the $(G'/G)$ expansion method^9,10^, the improved generalized Riccati equation mapping method^11^. The study of propagation across optical fibers frequently makes use of the non-linear Schrödinger equation (NLSE)^12–20^. The impact of noise on various dynamical models is a significant study domain within the applied sciences, particularly in physics. Stochastic partial differential equations (SPDEs) are considered as a mathematical tool for analyzing systems with unanticipated outcomes^21–24^. These equations may model a broad range of stochastic dynamics in complex systems, whether naturally occurring or artificially constructed. The theory of SPDEs is based on modern stochastic analysis and the science of deterministic partial differential equations. It is possible to extract efficient solutions for NPDEs, which can aid in the understanding of several physical processes. In order to explain how pulses propagate in optical fibers, several nonlinear PDEs exist. The models that are most frequently employed include the Kudryashov equation^25^, and three other equations: Triki–Biswas^26^, Biswas–Arshed^27^, and Fokas–Lenells^28^. Academic researchers have all studied these. Many other models grabbed the attention of many scientists to study them to create novel optical soliton solutions^29,32–36^.

In the current piece of work, an equation of high order that exhibits nonlinearity with weak non-local effects is known as SNLSE. This equation gives rise to optical solitons^29^ which can be read as:

1 $$\begin{aligned} & i \mathcal {R}_t+i \eta _1 \mathcal {R}_{xxx}+\eta _2 \mathcal {R}_{xxxx}+\mathcal {R} \left( \eta _3 \left| \mathcal {R} \right| ^2+\eta _4 \left| \mathcal {R} \right| ^4+\eta _5 \left( \left| \mathcal {R} \right| ^2\right) _{xx}\right) -i \left( \eta _6 \mathcal {R} _x+\eta _7 \left( \mathcal {R} \left| \mathcal {R} \right| ^2\right) _x+\eta _8 \mathcal {R} \left( \left| \mathcal {R} \right| ^2\right) _x\right) \nonumber \\ & +\varrho \ \mathcal {R}\ W_t=0, \end{aligned}$$

where $i=\sqrt{-1}$. In Eq. (1), $\mathcal {R} (x,t)$ denotes a complex envelope function, while the first term represents linear evolution of the time, $\eta _1$ and $\eta _2$ representing third and fourth order dispersions, respectively, while $\eta _3$ and $\eta _4$ represent parabolic nonlinearity, respectively. In the middle, $\eta _5$ represents non-local nonlinearity, $\eta _6$ represents inter-modal dispersion, $\eta _7$ depicts the effect of self-steepening, and $\eta _8$ acts as a nonlinear dispersion coefficient. Here, $\varrho$ is the noise intensity coefficient, while *W*(*t*) denotes a one-dimensional standard Wiener process. The final term in Eq. (1) represents stochastic forcing corresponding to Gaussian white noise via the Wiener process increment. Throughout this work, the model is formulated under the Itô interpretation.

Readers should be aware that^30^ retrieved novel solitons and additional solutions utilizing the unified Riccati expansion approach for the model specified in Eq. (1) in its deterministic version. The deterministic variant of the novel Kudryashov approach was used by Hosseini et al.^31^ to get soliton solutions of particular situations of Eq. (1).

By applying the modified extended mapping technique (MEMM), the optical soliton and exact solutions to the SNLSE are discovered. The originality of this work is strengthened by the application of this method, which has never been utilized to analyze this model before.

The rest of the paper is organized as follows. Section “Preliminaries of the applied technique” presents the outline of the proposed method. The obtained analytical results are discussed in Sect. “Nonlinear stochastic waves”. Section “White noise influence on some retrieved solitons” presents graphical illustrations of the solutions in both two- and three-dimensional plots. Section “Linear Stability Analysis” provides a rigorous linear stability analysis of the obtained solutions. Finally, the concluding remarks are given in Sect. “Conclusion”.

## Preliminaries of the applied technique

The MEMM is briefly discussed in this section. The MEMM^1,15,36^, one of the more modern approaches, has drawn a lot of attention due to its potential for handling NPDEs. Consider the following NPDE:

2 $$\begin{aligned} \mathcal {F}(\mathfrak {q},\mathfrak {q}_x,\mathfrak {q}_t,\mathfrak {q}_{xt},\mathfrak {q}_{xx},\mathfrak {q}_{xxx},....)=0. \end{aligned}$$

To solve Eq. (2), use the algorithmic techniques outlined below:

**Step-(1)**: Using the wave transformation that follows:

3 $$\begin{aligned} \mathfrak {q}(x,t)=\mathfrak {Q}(\zeta ); ~ \zeta =x-\nu t,~ \nu \ne 0, \end{aligned}$$

an ordinary differential equation (ODE) can be created from the NPDE in Eq. (2). In which, $\nu$ represents the speed of the wave. This ODE can be read as:

4 $$\begin{aligned} \mathcal {H}(\mathfrak {Q},\mathfrak {Q}',\mathfrak {Q}'',\mathfrak {Q}''',....)=0 \end{aligned}$$

**Step-(2)**: According to this approach, the solution of Eq. (4) can be expressed as:

5 $$\begin{aligned} \mathfrak {Q}(\zeta )=\sum _{j=0}^{N}{a_j \hbar ^j(\zeta )}+\sum _{j=-1}^{-N}{b_{-j} \hbar ^j(\zeta )}+\sum _{j=2}^{N}{c_j \hbar ^{j-2}(\zeta )\hbar '(\zeta )}+\sum _{j=-1}^{-N}{d_{-j} \hbar ^{j}(\zeta )\hbar '(\zeta )}, \end{aligned}$$

where the function $\hbar (\zeta )$ must meet the following requirements, and $a_j, b_{-j}, c_j,$ and $d_{-j}$ are constants with real values that need to be assessed:

6 $$\begin{aligned} \hbar '(\zeta )=\sqrt{\iota _0+\iota _1 \hbar (\zeta )+\iota _2 \hbar ^2(\zeta )+\iota _3 \hbar ^3(\zeta )+\iota _4 \hbar ^4(\zeta )+\iota _6 \hbar ^6(\zeta )} \end{aligned}$$

where the constant values $\iota _i,\ (i = 0, 1, 2, 3, 4, 6)$ will result in several types of solutions.

**Step-(3)**: The balancing concept is used in Eq. (4) to obtain the integer *N*.

**Step-(4)**:The hypothesized solution from Eqs. (5) and (6) may be inserted into Eq. (4) to get a system of equations in the unknowns $a_j,b_{-j},c_j,d_{-j},\nu$. The terms of the coefficients of $\hbar '^j(\zeta )\hbar ^i(\zeta )$ ($j=0,1$); It is therefore possible to equalize $i=0,\pm 1,\pm 2,...$ to zero. Use the Wolfram Mathematica^®^ package to handle the resulting system raised. After establishing that, we may determine the constants $a_j,b_{-j},c_j,d_{-j},\nu$.

**Step-(5)**: By choosing various values for $\iota _0,\iota _1,\iota _2,\iota _3,\iota _4,\iota _6$, Eq. (6) might yield a variety of exact solutions, including the following:

**Case 1:** For $$\iota _0=\iota _1=\iota _3=\iota _6=0$$,

$$\begin{aligned} \hbar (\zeta )=\sqrt{-\frac{\iota _2}{\iota _4}} \text {sech}\left( \sqrt{\iota _2} \zeta \right) ,\quad \iota _2>0, \iota _4<0. \end{aligned}$$

$$\begin{aligned} \hbar (\zeta )=\sqrt{-\frac{\iota _2}{\iota _4}} \sec \left( \sqrt{-\iota _2} \zeta \right) , \quad \iota _2<0, \iota _4>0. \end{aligned}$$

$$\begin{aligned} \hbar (\zeta )=\sqrt{-\frac{\iota _2}{\iota _4}} \csc \left( \sqrt{-\iota _2} \zeta \right) , \quad \iota _2<0, \iota _4>0. \end{aligned}$$

**Case 2:** For $$\iota _1=\iota _3=\iota _6=0, \iota _0=\dfrac{\iota _2^2}{4\iota _4}$$,

$$\begin{aligned} \hbar (\zeta )=\sqrt{-\frac{\iota _2}{2 \iota _4}} \tanh \left( \sqrt{-\frac{\iota _2}{2}} \zeta \right) ,\quad \iota _2<0, \iota _4>0. \end{aligned}$$

$$\begin{aligned} \hbar (\zeta )=\sqrt{\frac{\iota _2}{2 \iota _4}} \tan \left( \sqrt{\frac{\iota _2}{2}} \zeta \right) ,\quad \iota _2>0, \iota _4>0. \end{aligned}$$

**Case 3:** For $$\iota _3=\iota _4=\iota _6=0$$,

$$\begin{aligned} \hbar (\zeta )=\frac{\iota _1 \sinh \left( 2 \sqrt{\iota _2} \zeta \right) }{2 \iota _2}-\frac{\iota _1}{2 \iota _2},\quad \iota _2>0,\iota _0=0. \end{aligned}$$

$$\begin{aligned} \hbar (\zeta )=\frac{\iota _1 \sin \left( \sqrt{-\iota _2} \zeta \right) }{2 \iota _2}-\frac{\iota _1}{2 \iota _2},\quad \iota _2<0,\iota _0=0. \end{aligned}$$

$$\begin{aligned} \hbar (\zeta )=\sqrt{\frac{\iota _0}{\iota _2}} \sinh \left( \sqrt{\iota _2} \zeta \right) ,\quad \iota _0>0,\iota _2>0,\iota _1=0. \end{aligned}$$

$$\begin{aligned} \hbar (\zeta )=\sqrt{-\frac{\iota _0}{\iota _2}} \sin \left( \sqrt{-\iota _2} \zeta \right) ,\quad \iota _0>0,\iota _2<0,\iota _1=0. \end{aligned}$$

$$\begin{aligned} \hbar (\zeta )=\exp \left( \sqrt{\iota _2} \zeta \right) -\frac{\iota _1}{2 \iota _2},\quad \iota _2>0,\iota _0=\frac{\iota _1^2}{4 \iota _2}. \end{aligned}$$

**Case 4:** For $$\iota _0=\iota _1=\iota _6=0$$,

$$\begin{aligned} \hbar (\zeta )=-\frac{\iota _2 \left( \tanh \left( \frac{1}{2} \sqrt{\iota _2} \zeta \right) +1\right) }{\iota _3},\quad \iota _3^2=4\iota _2\iota _4,\iota _2>0. \end{aligned}$$

$$\begin{aligned} \hbar (\zeta )=-\frac{\iota _2 \left( \coth \left( \frac{1}{2} \sqrt{\iota _2} \zeta \right) +1\right) }{\iota _3},\quad \iota _3^2=4\iota _2\iota _4,\iota _2>0. \end{aligned}$$

$$\begin{aligned} \hbar (\zeta )=\frac{\iota _2 \text {sech}^2\left( \frac{1}{2} \sqrt{\iota _2} \zeta \right) }{2 \sqrt{\iota _2 \iota _4} \tanh \left( \frac{1}{2} \sqrt{\iota _2} \zeta \right) -\iota _3},\quad \iota _3^2\ne 4\iota _2\iota _4,\iota _2>0,\iota _4>0. \end{aligned}$$

$$\begin{aligned} \hbar (\zeta )=-\frac{\iota 2 \sec ^2\left( \frac{1}{2} \sqrt{-\iota _2}\zeta \right) }{2 \sqrt{-\iota _2 \iota _4} \tan \left( \frac{1}{2} \sqrt{-\iota _2} \zeta \right) +\iota _3},\quad \iota _3^2\ne 4\iota _2\iota _4,\iota _2<0,\iota _4>0. \end{aligned}$$

**Case 5:** For $$\iota _2=\iota _4=\iota _6=0$$,

$$\begin{aligned} \hbar (\zeta )=\wp \left( \frac{1}{2} \sqrt{\iota _3}\zeta ;-\frac{4 \iota _1}{\iota _3},-\frac{4 \iota _0}{\iota _3}\right) ,\quad \iota _3>0. \end{aligned}$$

**Case 6:** For $$\iota _1=\iota _3=0$$,

$$\begin{aligned} \hbar (\zeta )=\sqrt{\frac{2 \iota _2 \text {sech}^2\left( \sqrt{\iota _2} \zeta \right) }{2 \sqrt{\iota _4^2-4 \iota _2 \iota _6}-\left( \sqrt{\iota _4^2-4 \iota _2 \iota _6}+\iota _4\right) \text {sech}^2\left( \sqrt{\iota _2}\zeta \right) }},\quad \iota _2>0. \end{aligned}$$

$$\begin{aligned} \hbar (\zeta )=\sqrt{\frac{2 \iota _2 \sec ^2\left( \sqrt{-\iota _2}\zeta \right) }{2 \sqrt{\iota _4^2-4 \iota _2 \iota _6}-\left( \sqrt{\iota _4^2-4 \iota _2 \iota _6}-\iota _4\right) \sec ^2\left( \sqrt{-\iota _2}\zeta \right) }},\quad \iota _2<0. \end{aligned}$$

**Step-(6)**: Upon substituting the established constants $a_j,b_{-j},c_j,d_{-j}$ through Eq. (5), including the results from Eq. (6), several solutions for Eq. One could obtain (2).

## Nonlinear stochastic waves

In this section of the study, the following transformation is employed to provide analytical, stochastic solutions for Eq. (1), which can expressed as:

7 $$\begin{aligned} \mathcal {R} (x,t)=\vartheta (\xi ) e^{i \left( -\beta x+\omega t-\varrho ^2 t + \varrho W(t) \right) },\qquad \xi =x-\nu t, \end{aligned}$$

with $\varrho , \omega$, and $\beta$ being real-valued constants.

When applying the described transformation in Eq. (7), Eq. (1) can produce two parts which are real and imaginary parts respectively, as below:

8 $$\begin{aligned} & \eta _4 \vartheta (\xi )^5+\eta _2 \vartheta (\xi )^{(4)}+\vartheta (\xi )^3 \left( \eta _3-\beta \eta _7\right) +\vartheta (\xi )'' \left( -6 \beta ^2 \eta _2+3 \beta \eta _1+2 \eta _5 \vartheta (\xi )^2\right) +2 \eta _5 \vartheta (\xi ) \left( \vartheta (\xi )'\right) ^2\nonumber \\ & +\left( \beta ^4 \eta _2-\beta ^3 \eta _1-\beta \eta _6-\omega +\varrho ^2\right) \vartheta (\xi ) =0, \end{aligned}$$

9 $$\begin{aligned} \left( \eta _1-4 \beta \eta _2\right) \vartheta (\xi )^{(3)} -\left( -4 \beta ^3 \eta _2+3 \beta ^2 \eta _1+\eta _6+\nu \right) \vartheta (\xi )' -\left( 3 \eta _7+2 \eta _8\right) \vartheta (\xi )^2 \vartheta (\xi )'=0. \end{aligned}$$

Equation (9) is utilized to get the soliton wave number, soliton velocity, and parameter $\eta _8$ using the linearly independent approach as:

10 $$\begin{aligned} \beta =\dfrac{\eta _1}{4 \eta _2},\quad \nu =-\dfrac{\eta _1^3+8 \eta _2^2 \eta _6}{8 \eta _2^2},\quad \eta _8=-\dfrac{3 \eta _7}{2}. \end{aligned}$$

Then, Eq. (8) reduces to

11 $$\begin{aligned} \eta _4 \vartheta ^5+\eta _2 \vartheta ^{(4)}+\eta _3 \vartheta ^3+2 \eta _5 \vartheta ^2 \vartheta ''+\frac{3 \eta _1^2 \vartheta ''}{8 \eta _2}-\frac{\eta _1 \vartheta \left( \eta _6+\eta _7 \vartheta ^2\right) }{4 \eta _2}+2 \eta _5 \vartheta \left( \vartheta '\right) ^2-\frac{3 \eta _1^4 \vartheta }{256 \eta _2^3}-\omega \vartheta +\varrho ^2\vartheta =0, \end{aligned}$$

To construct an exact solution of Eq. (11), we first perform the standard balancing procedure. By balancing the highest-order derivative term, $\vartheta ^{(4)}$, with the highest-order nonlinear term, $\vartheta ^5$, we find that the appropriate solution ansatz requires $N = 1$. It is important to note that this procedure and the resulting solutions are valid only for $\eta _4 \ne 0$. When $\eta _4 = 0$, Eq. (11) reduces to a stochastic NLSE with Kerr-type nonlinearity, which requires a distinct balance and solution structure and is therefore not covered by the present analysis. Accordingly, the expression for the solution of Eq. (11) can be written as

12 $$\begin{aligned} \vartheta (\xi )={a}_0+{a}_1 \hbar (\xi )+\frac{{b}_1}{\hbar (\xi )}+\frac{{d}_1 \hbar '(\xi )}{\hbar (\xi )}. \end{aligned}$$

By substituting Eq. (12) and Eq. (6) into Eq. (11) and aggregating all coefficients of $\hbar ^{j}$ to zero, Wolfram Mathematica can resolve a system of equations yielding the following results:

**Set-(1)**: If $\iota _0=\iota _1=\iota _3=\iota _6=0$, then we have

**Result 1**:

$$a_0=b_1=d_1=0, \hspace{0.2cm} a_1=-\sqrt{-\frac{\sqrt{3 \left( 3 \eta _5^2-8 \eta _2 \eta _4\right) }+3 \eta _5}{\eta _4}} \iota _4,\hspace{0.2cm} \iota _2=-\frac{T}{16 \eta _2^4}.$$

Then, we get

13 $$\begin{aligned} & \mathcal {R}(x,t)=\left( -\frac{\sqrt{-\frac{\left( \sqrt{3 \left( 3 \eta _5^2-8 \eta _2 \eta _4\right) }+3 \eta _5\right) T}{\eta _4}}}{4 \eta _2^2}\text {sech}\left( \frac{\sqrt{-T}}{4 \eta _2^2}(x-\nu t)\right) \right) e^{i \left( -\beta x+\omega t-\varrho ^2 t + \varrho W(t) \right) },\nonumber \\ & \hspace{0.2cm}\eta _4<0, \hspace{0.2cm}\eta _2>0,\hspace{0.2cm} T<0. \end{aligned}$$

14 $$\begin{aligned} & \mathcal {R}(x,t)=\left( -\frac{\sqrt{-\frac{\left( \sqrt{3 \left( 3 \eta _5^2-8 \eta _2 \eta _4\right) }+3 \eta _5\right) T}{\eta _4}} }{4 \eta _2^2}\sec \left( \frac{\sqrt{T} }{4 \eta _2^2}(x-\nu t)\right) \right) e^{i \left( -\beta x+\omega t-\varrho ^2 t + \varrho W(t) \right) },\nonumber \\ & \hspace{0.2cm}\eta _4<0, \hspace{0.2cm}\eta _2>0,\hspace{0.2cm} T>0. \end{aligned}$$

15 $$\begin{aligned} & \mathcal {R}(x,t)=\left( -\frac{\sqrt{-\frac{\left( \sqrt{3 \left( 3 \eta _5^2-8 \eta _2 \eta _4\right) }+3 \eta _5\right) T}{ \eta _4}} }{4 \eta _2^2}\csc \left( \frac{\sqrt{T} }{4 \eta _2^2}(x-\nu t)\right) \right) e^{i \left( -\beta x+\omega t-\varrho ^2 t + \varrho W(t) \right) },\nonumber \\ & \hspace{0.2cm}\eta _4<0, \hspace{0.2cm}\eta _2>0,\hspace{0.2cm} T>0. \end{aligned}$$

where $$T=3 \eta _1^2 \eta _2^2+2 \sqrt{64 \eta _2^7 \left( \omega -\varrho ^2\right) +16 \eta _1 \eta _6 \eta _2^6+3 \eta _1^4 \eta _2^4}$$.

While Eqs. (14) and (15) are stochastic singular periodic solutions, Eq. (13) defines a stochastic bright soliton.

**Result 2**:

$$a_0=b_1=d_1=0, \hspace{0.2cm}a_1=-\sqrt{-\frac{\sqrt{3 \left( 3 \eta _5^2-8 \eta _2 \eta _4\right) }+3 \eta _5}{\eta _4}} \iota _4,\hspace{0.2cm} \iota _2=\frac{R}{16 \eta _2^4}.$$

Then, we get

16 $$\begin{aligned} & \mathcal {R}(x,t)=\left( -\frac{\sqrt{\frac{\left( \sqrt{3 \left( 3 \eta _5^2-8 \eta _2 \eta _4\right) }+3 \eta _5\right) R}{\eta _4}} }{4 \eta _2^2}\text {sech}\left( \frac{\sqrt{R} }{4 \eta _2^2}(x-\nu t)\right) \right) e^{i \left( -\beta x+\omega t-\varrho ^2 t + \varrho W(t) \right) }, \nonumber \\ & \hspace{0.2cm}R>0, \hspace{0.2cm}\eta _2>0, \hspace{0.2cm}\eta _4>0. \end{aligned}$$

17 $$\begin{aligned} & \mathcal {R}(x,t)=\left( -\frac{\sqrt{\frac{\left( \sqrt{3 \left( 3 \eta _5^2-8 \eta _2 \eta _4\right) }+3 \eta _5\right) R}{\eta _4}} }{4 \eta _2^2}\sec \left( \frac{\sqrt{-R} }{4 \eta _2^2}(x-\nu t)\right) \right) e^{i \left( -\beta x+\omega t-\varrho ^2 t + \varrho W(t) \right) },\nonumber \\ & \hspace{0.2cm}\eta _2>0, \hspace{0.2cm}R<0, \hspace{0.2cm}\eta _4>0. \end{aligned}$$

18 $$\begin{aligned} & \mathcal {R}(x,t)=\left( -\frac{\sqrt{\frac{\left( \sqrt{3 \left( 3 \eta _5^2-8 \eta _2 \eta _4\right) }+3 \eta _5\right) R}{\eta _4}} }{4 \eta _2^2}\csc \left( \frac{\sqrt{-R} }{4 \eta _2^2}(x-\nu t)\right) \right) e^{i \left( -\beta x+\omega t-\varrho ^2 t + \varrho W(t) \right) },\nonumber \\ & \hspace{0.2cm}\eta _4>0, \hspace{0.2cm}\eta _2>0, \hspace{0.2cm}R<0. \end{aligned}$$

where $$R=2 \sqrt{64 \eta _2^7 \left( \omega -\varrho ^2\right) +16 \eta _1 \eta _6 \eta _2^6+3 \eta _1^4 \eta _2^4}-3 \eta _1^2 \eta _2^2.$$

While Eqs. (17) and (18) are stochastic singular periodic solutions, Eq. (16) depicts a stochastic bright soliton.

**Set-(2)**: If $\iota _{1}=\iota _{3}=\iota _{5}=0,\hspace{0.3cm}\iota _{0}=\frac{\iota _{2}^2}{4 \iota _{4}}$, then we have

$$a_0=d_1=b_1=0,\hspace{0.2cm}a_1=-\sqrt{\frac{\mu }{\eta _4}},\hspace{0.2cm}\iota _2=\frac{\sqrt{2} \sigma -3 \eta _1^2 \eta _2^2 \left( 8 \eta _2 \eta _4 \iota _4+\eta _5 \left( \sqrt{3} Q-3 \eta _5 \iota _4\right) \right) }{64 \eta _2^4 \left( 8 \eta _2 \eta _4-3 \eta _5^2\right) \iota _4}$$.

Then, we get

19 $$\begin{aligned} & \mathcal {R}(x,t)= \left( \frac{1}{8}\sqrt{\frac{\mu \gamma }{2\eta _4}} \tanh \left( \frac{1}{8}\sqrt{-\frac{\sqrt{2} \sigma -3 \eta _1^2 \eta _2^2 \left( 8 \eta _2 \eta _4 \iota _4+\eta _5 \mu \right) }{2\eta _2^4 \left( 8 \eta _2 \eta _4-3 \eta _5^2\right) \iota _4}}(x-\nu t)\right) \right) e^{i \left( -\beta x+\omega t-\varrho ^2 t + \varrho W(t) \right) },\nonumber \\ & \gamma>0,\hspace{0.2cm}\mu>0,\hspace{0.2cm}\eta _4>0,\hspace{0.2cm}\sqrt{2} \sigma -3 \eta _1^2 \eta _2^2 \left( 8 \eta _2 \eta _4 \iota _4+\eta _5 \mu \right) >0,\hspace{0.2cm}\iota _4<0. \end{aligned}$$

20 $$\begin{aligned} & \mathcal {R}(x,t)=\left( \frac{1}{8}\sqrt{\frac{\mu \gamma }{2\eta _4}} \tan \left( \frac{1}{8}\sqrt{\frac{\sqrt{2} \sigma -3 \eta _1^2 \eta _2^2 \left( 8 \eta _2 \eta _4 \iota _4+\eta _5 \mu \right) }{2\eta _2^4 \left( 8 \eta _2 \eta _4-3 \eta _5^2\right) \iota _4}}(x-\nu t)\right) \right) e^{i \left( -\beta x+\omega t-\varrho ^2 t + \varrho W(t) \right) }, \nonumber \\ & \gamma>0,\hspace{0.2cm}\mu>0,\hspace{0.2cm}\eta _4>0,\hspace{0.2cm}\sqrt{2} \sigma -3 \eta _1^2 \eta _2^2 \left( 8 \eta _2 \eta _4 \iota _4+\eta _5 \mu \right)>0,\hspace{0.2cm}\iota _4>0. \end{aligned}$$

where $$Q=\sqrt{\left( 3 \eta _5^2-8 \eta _2 \eta _4\right) \iota _4^2},\hspace{0.3cm}\mu =\sqrt{3} Q-3 \eta _5 \iota _4$$,

$$\rho =8 \eta _2 \eta _4 \iota _4+\eta _5 \mu$$,

$$\sigma =\sqrt{-\eta _2^4 \left( 8 \eta _2 \eta _4-3 \eta _5^2\right) \iota _4 \left( -3 \eta _1^4 \left( 28 \eta _2 \eta _4 \iota _4+5 \eta _5 \mu \right) +512 \eta _2^3 \rho \left( \varrho ^2-\omega \right) -128 \eta _2^2 \eta _6 \eta _1 \rho \right) }$$,

$$\gamma =\sqrt{\sqrt{2} \sigma -\frac{3 \eta _1^2 \eta _2^2 \rho }{\eta _2^4 \left( 8 \eta _2 \eta _4-3 \eta _5^2\right) \iota _4^2}}$$.

Whereas the previous stochastic solutions are of types: Eq. (19) is a dark soliton solution, and Eq. (20) is a single periodic solution.

**Set-(3)**:

(i) If $\iota _{0}=\iota _{3}=\iota _{4}=\iota _{6}=0$, then we have

**Result 1**:

$$\begin{aligned} & b_1=\eta _4=d_1=0,\hspace{0.2cm}a_1=\frac{a_0 \left( \eta _1 \eta _7-4 \eta _2 \eta _3\right) }{8 \eta _2 \eta _5 \iota _1},\hspace{0.2cm}\iota _2=\frac{\eta _1 \eta _7-4 \eta _2 \eta _3}{16 \eta _2 \eta _5}. \end{aligned}$$

One can estimate the upcoming hyperbolic and trigonometric (periodic) solutions, respectively:

21 $$\begin{aligned} & \mathcal {R}(x,t)=a_0 \sinh \left( \frac{1}{2} \sqrt{\frac{\eta _1 \eta _7-4 \eta _2 \eta _3}{\eta _2 \eta _5}} (x-\nu t)\right) e^{i \left( -\beta x+\omega t-\varrho ^2 t + \varrho W(t) \right) }, \end{aligned}$$

such that $$(\eta _1 \eta _7-4 \eta _2 \eta _3)(\eta _2 \eta _5)>0$$.

22 $$\begin{aligned} & \mathcal {R}(x,t)=a_0 \sin \left( \frac{1}{2} \sqrt{\frac{4 \eta _2 \eta _3-\eta _1 \eta _7}{\eta _2 \eta _5}} (x-\nu t)\right) e^{i \left( -\beta x+\omega t-\varrho ^2 t + \varrho W(t) \right) }, \end{aligned}$$

such that $$(\eta _1 \eta _7-4 \eta _2 \eta _3)(\eta _2 \eta _5)<0$$.

**Result 2**:

$$\begin{aligned} & b_1=d_1=\eta _4=0,\hspace{0.2cm}a_0=\frac{\sqrt{\frac{\delta +3 \eta _5^2 \eta _1^4-6 \eta _2 \eta _5 \eta _7 \eta _1^3-16 \eta _2^4 \eta _3^2+\lambda }{4 \eta _2 \eta _3-\eta _1 \eta _7}}}{4 \eta _2 \eta _5},\hspace{0.2cm}\iota _2=\frac{\eta _1 \eta _7-4 \eta _2 \eta _3}{16 \eta _5 \eta _2},\nonumber \\ & a_1=\frac{\left( \eta _1 \eta _7-4 \eta _2 \eta _3\right) \sqrt{\delta +3 \eta _5^2 \eta _1^4-6 \eta _2 \eta _5 \eta _7 \eta _1^3-16 \eta _2^4 \eta _3^2+\lambda }}{32 \sqrt{2} \eta _2 \eta _5 \sqrt{\eta _2^2 \eta _5^2 \left( 4 \eta _2 \eta _3-\eta _1 \eta _7\right) } \iota _1}. \end{aligned}$$

One can estimate the upcoming hyperbolic and trigonometric (periodic) solutions, respectively:

23 $$\begin{aligned} & \mathcal {R}(x,t)=\frac{\sqrt{\frac{\delta +3 \eta _5^2 \eta _1^4-6 \eta _2 \eta _5 \eta _7 \eta _1^3-16 \eta _2^4 \eta _3^2+\lambda }{2(4 \eta _2 \eta _3-\eta _1 \eta _7)}}}{4 \eta _2 \eta _5}\sinh \left( \frac{1}{2} \sqrt{\frac{\eta _1 \eta _7-4 \eta _2 \eta _3}{\eta _2 \eta _5}} (x-\nu t)\right) \nonumber \\ & \times e^{i \left( -\beta x+\omega t-\varrho ^2 t + \varrho W(t) \right) }, \end{aligned}$$

such that $$(\eta _1 \eta _7-4 \eta _2 \eta _3)(\eta _2 \eta _5)>0$$.

24 $$\begin{aligned} & \mathcal {R}(x,t)=\frac{\sqrt{\frac{\delta +3 \eta _5^2 \eta _1^4-6 \eta _2 \eta _5 \eta _7 \eta _1^3-16 \eta _2^4 \eta _3^2+\lambda }{2(4 \eta _2 \eta _3-\eta _1 \eta _7)}}}{4 \eta _2 \eta _5}\sin \left( \frac{1}{2} \sqrt{\frac{4 \eta _2 \eta _3-\eta _1 \eta _7}{\eta _2 \eta _5}} (x-\nu t)\right) \nonumber \\ & \times e^{i \left( -\beta x+\omega t-\varrho ^2 t + \varrho W(t) \right) }, \end{aligned}$$

such that $$(4 \eta _2 \eta _3-\eta _1 \eta _7)( \eta _2 \eta _5)>0.$$

where $$\delta =8 \eta _2^3 \left( \eta _1 \eta _3 \eta _7-32 \eta _5^2 \left( \varrho ^2-\omega \right) \right) ,\hspace{0.2cm}\lambda =\eta _1 \eta _2^2 \left( 64 \eta _6 \eta _5^2+\eta _1 \left( 24 \eta _3 \eta _5-\eta _7^2\right) \right)$$.

(ii) If $\iota _{1}=\iota _{3}=\iota _{4}=\iota _{6}=0$, then we have

**Result 1**:

$$\begin{aligned} a_0=\eta _4=d_1=0,\hspace{0.2cm}a_1=\frac{-3 \eta _5 \eta _1^2-5 \eta _2 \eta _7 \eta _1+20 \eta _2^2 \eta _3}{24 b_1 \eta _2 \eta _5^2},\hspace{0.2cm}\iota _0=-\frac{b_1^2 \eta _5}{4 \eta _2},\hspace{0.2cm}\iota _2=\frac{\eta _1 \eta _7-4 \eta _2 \eta _3}{16 \eta _2 \eta _5}. \end{aligned}$$

Then, we get

25 $$\begin{aligned} & \mathcal {R}(x,t)=\left( \frac{b_1 \left( \frac{\left( -3 \eta _5 \eta _1^2-5 \eta _2 \eta _7 \eta _1+20 \eta _2^2 \eta _3\right) \sinh \left( \frac{1}{4} \sqrt{\frac{\eta _1 \eta _7-4 \eta _2 \eta _3}{\eta _2 \eta _5}} (x-\nu t)\right) }{4 \eta _2^2 \eta _3-\eta _1 \eta _2 \eta _7}+6 \text {csch}\left( \frac{1}{4} \sqrt{\frac{\eta _1 \eta _7-4 \eta _2 \eta _3}{\eta _2 \eta _5}} (x-\nu t)\right) \right) }{12 \sqrt{\frac{b_1^2 \eta _5^2}{4 \eta _2 \eta _3-\eta _1 \eta _7}}}\right) \nonumber \\ & \times e^{i \left( -\beta x+\omega t-\varrho ^2 t + \varrho W(t) \right) }, \end{aligned}$$

such that $\eta _1>0,\hspace{0.2cm}\eta _3>0,\hspace{0.2cm}\eta _5>0,$ and $\hspace{0.2cm}\eta _7>0,$ while $\eta _2<0$.

26 $$\begin{aligned} & \mathcal {R}(x,t)=\left( \frac{b_1 \left( \frac{\left( 3 \eta _5 \eta _1^2+5 \eta _2 \eta _7 \eta _1-20 \eta _2^2 \eta _3\right) \sin \left( \frac{1}{4} \sqrt{\frac{4 \eta _2 \eta _3-\eta _1 \eta _7}{\eta _2 \eta _5}} (x-\nu t)\right) }{\eta _2 \left( 4 \eta _2 \eta _3-\eta _1 \eta _7\right) }+6 \csc \left( \frac{1}{4} \sqrt{\frac{4 \eta _2 \eta _3-\eta _1 \eta _7}{\eta _2 \eta _5}} (x-\nu t)\right) \right) }{12 \sqrt{\frac{b_1^2 \eta _5^2}{\eta _1 \eta _7-4 \eta _2 \eta _3}}}\right) \nonumber \\ & \times e^{i \left( -\beta x+\omega t-\varrho ^2 t + \varrho W(t) \right) }, \end{aligned}$$

such that $\eta _1>0, \hspace{0.2cm}\eta _2>0,\hspace{0.2cm}\eta _3>0,$ and $\eta _5>0,$ while $\eta _7<0.$ Equation (26) provides a singular periodic solution, but Eq. (25) is a singular soliton.

**Result 2**:

$$\begin{aligned} & a_0=b_1=\eta _4=d_1=0,\hspace{0.2cm}\iota _2=\frac{\eta _1 \eta _7-4 \eta _2 \eta _3}{16 \eta _2 \eta _5},\nonumber \\ & \omega =\frac{8 \eta _2^3 \left( 64 a_1^2 \eta _5^3 \iota _0+32 \eta _5^2 \varrho ^2-\eta _1 \eta _3 \eta _7\right) -3 \eta _5^2 \eta _1^4+6 \eta _2 \eta _5 \eta _7 \eta _1^3+\eta _2^2 \left( \eta _1 \left( \eta _7^2-24 \eta _3 \eta _5\right) -64 \eta _5^2 \eta _6\right) \eta _1+16 \eta _2^4 \eta _3^2}{256 \eta _2^3 \eta _5^2}. \end{aligned}$$

One can estimate the upcoming hyperbolic and trigonometric (periodic) solutions, respectively:

27 $$\begin{aligned} & \mathcal {R}(x,t)=\left( 4 a_1 \sqrt{\frac{\eta _2 \eta _5 \iota _0}{\eta _1 \eta _7-4 \eta _2 \eta _3}} \sinh \left( \frac{1}{4} \sqrt{\frac{\eta _1 \eta _7-4 \eta _2 \eta _3}{\eta _2 \eta _5}} (x-\nu t)\right) \right) \nonumber \\ & \times e^{i \left( -\beta x+\omega t-\varrho ^2 t + \varrho W(t) \right) }, \end{aligned}$$

such that $\eta _1>0,\hspace{0.2cm}\eta _3>0,\hspace{0.2cm}\eta _5>0,$ and $\eta _7>0$ while $\eta _2<0$.

28 $$\begin{aligned} & \mathcal {R}(x,t)=\left( 4 a_1 \sqrt{\frac{\eta _2 \eta _5 \iota _0}{4 \eta _2 \eta _3-\eta _1 \eta _7}} \sin \left( \frac{1}{4} \sqrt{\frac{4 \eta _2 \eta _3-\eta _1 \eta _7}{\eta _2 \eta _5}} (x-\nu t)\right) \right) \nonumber \\ & \times e^{i \left( -\beta x+\omega t-\varrho ^2 t + \varrho W(t) \right) }, \end{aligned}$$

such that $\eta _1>0, \hspace{0.2cm}\eta _2>0,\hspace{0.2cm}\eta _3>0,$ and $\eta _5>0,$ while $\eta _7<0.$

**Set-(4)**: $\iota _0=\iota _1=\iota _2=\iota _6=0$, then we obtain

$$\begin{aligned} & b_1=d_1=0,\hspace{0.2cm}\omega =-\frac{9 \eta _1^4}{256 \eta _2^3}-\frac{\eta _6 \eta _1}{4 \eta _2}+\varrho ^2,\hspace{0.2cm}\eta _4=\frac{3 \eta _1^4}{128 a_0^4 \eta _2^3},\hspace{0.2cm}\eta _5=-\frac{5 \eta _1^2}{16 a_0^2 \eta _2},\hspace{0.2cm}\eta _3=\frac{\eta _1 \eta _7}{4 \eta _2}-\frac{3 \eta _1^4}{64 a_0^2 \eta _2^3}\nonumber \\ & a_1=\frac{4 a_0 \eta _2^2 \iota _3}{\eta _1^2},\hspace{0.2cm}\iota _4=\frac{a_1^2 \eta _1^2}{16 a_0^2 \eta _2^2}. \end{aligned}$$

Then, we obtain

29 $$\begin{aligned} \mathcal {R}(x,t)=\left( \frac{a_0 \left( 12 \eta _2^2+\eta _1^2 (x-\nu t)^2\right) }{\eta _1^2 (x-\nu t)^2-4 \eta _2^2}\right) e^{i \left( -\beta x+\omega t-\varrho ^2 t + \varrho W(t) \right) }, \hspace{0.2cm}\eta _1^2 (x-\nu t)^2-4 \eta _2^2\ne 0. \end{aligned}$$

Eq. (29) represents a stochastic rational solution.

**Set-(5)**: If $\iota _0=\iota _1=\iota _6=0$, then we obtain

$$\begin{aligned} a_0=b_1=d_1=\iota _3=0,\hspace{0.2cm}a_1=\sqrt{\frac{\sqrt{3} \delta -3 \eta _5 \iota _4}{\eta _4}},\hspace{0.2cm}\iota _2=\frac{\aleph -\mathcal {H}}{16 \eta _2^2 \left( 25 \eta _2 \eta _4-6 \eta _5^2\right) \iota _4}. \end{aligned}$$

Then, we get

30 $$\begin{aligned} & \mathcal {R}(x,t)= \left( \frac{\sqrt{\frac{(\aleph -\mathcal {H}) \left( \sqrt{3} \delta -3 \eta _5 \iota _4\right) }{\eta _4 \left( 25 \eta _2 \eta _4-6 \eta _5^2\right) }}}{4 \iota _4 \eta _2} \text {csch}\left( \frac{1}{8} \sqrt{\frac{\aleph -\mathcal {H}}{\eta _2^2 \left( 25 \eta _2 \eta _4-6 \eta _5^2\right) \iota _4}}(x-\nu t)\right) \right) e^{i \left( -\beta x+\omega t-\varrho ^2 t + \varrho W(t) \right) },\nonumber \\ & \mathcal {H}<0, \hspace{0.2cm}\aleph>0,\hspace{0.2cm}\iota _4>0,\hspace{0.2cm}25 \eta _2 \eta _4-6 \eta _5^2>0. \end{aligned}$$

31 $$\begin{aligned} & \mathcal {R}(x,t)=\left( -\frac{\sqrt{\frac{(\mathcal {H}-\aleph ) \left( \sqrt{3} \delta -3 \eta _5 \iota _4\right) }{\eta _4 \left( 25 \eta _2 \eta _4-6 \eta _5^2\right) }} }{4 \iota _4 \eta _2}\csc \left( \frac{1}{8} \sqrt{\frac{\mathcal {H}-\aleph }{ \eta _2^2\left( 25 \eta _2 \eta _4-6 \eta _5^2\right) \iota _4}}(x-\nu t)\right) \right) e^{i \left( -\beta x+\omega t-\varrho ^2 t + \varrho W(t) \right) },\nonumber \\ & \mathcal {H}<0, \hspace{0.2cm}\aleph>0,\hspace{0.2cm}\iota _4>0,\hspace{0.2cm}25 \eta _2 \eta _4-6 \eta _5^2>0. \end{aligned}$$

where $$\delta =\sqrt{\left( 3 \eta _5^2-8 \eta _2 \eta _4\right) \iota _4^2},\hspace{0.2cm}\aleph =\eta _1 \eta _2 \eta _7 \left( 5 \sqrt{3} \delta +9 \eta _5 \iota _4\right) -4 \eta _2^2 \eta _3 \left( 5 \sqrt{3} \delta +9 \eta _5 \iota _4\right) ,$$

$$\mathcal {H}=3 \eta _1^2 \left( 5 \eta _2 \eta _4 \iota _4-\eta _5 \left( \sqrt{3} \delta +3 \eta _5 \iota _4\right) \right) .$$

Equation (31) provides a stochastic singular periodic solution, whereas Eq. (30) is a stochastic singular soliton.

**Set-(6)**: If $\iota _1=\iota _3=0$, then we obtain

$$\begin{aligned} & a_0=d_1=b_1=\iota _6=0,\hspace{0.2cm}\omega =\frac{1}{2} \sqrt{3} \iota _0 \sqrt{a_1^4 \left( 3 \eta _5^2-8 \eta _2 \eta _4\right) }+\frac{1}{2} a_1^2 \eta _5 \iota _0+\frac{3 \eta _1^2 \iota _2}{8 \eta _2}+\eta _2 \iota _2^2-\frac{3 \eta _1^4}{256 \eta _2^3}-\frac{\eta _6 \eta _1}{4 \eta _2}+\varrho ^2,\nonumber \\ & \eta _7=\frac{3 a_1^2 \left( 16 \eta _2^2 \left( 3 \eta _5 \iota _2+2 \eta _3\right) -3 \eta _1^2 \eta _5\right) +\sqrt{3} \sqrt{-a_1^4 \left( 8 \eta _2 \eta _4-3 \eta _5^2\right) } \left( 80 \eta _2^2 \iota _2+3 \eta _1^2\right) }{24 a_1^2 \eta _1 \eta _2},\nonumber \\ & \iota _4=\frac{\sqrt{3} \sqrt{a_1^4 \left( 3 \eta _5^2-8 \eta _2 \eta _4\right) }-3 a_1^2 \eta _5}{24 \eta _2}. \end{aligned}$$

Then, we get

32 $$\begin{aligned} & \mathcal {R}(x,t)=\left( a_1 \sqrt{\frac{2\iota _2 \text {sech}^2\left( \sqrt{\iota _2} (x-\nu t)\right) }{\frac{\varkappa }{12 \eta _2}-\left( \frac{\varkappa }{24 \eta _2}+\frac{\varkappa }{24 \eta _2}\right) \text {Sech}^2\left( \sqrt{\iota _2} (x-\nu t)\right) }}\right) e^{i \left( -\beta x+\omega t-\varrho ^2 t + \varrho W(t) \right) },\nonumber \\ & \iota _2>0,\hspace{0.2cm}\iota _6>0,\hspace{0.2cm}\varkappa >0. \end{aligned}$$

33 $$\begin{aligned} & \mathcal {R}(x,t)=\left( a_1 \sqrt{\frac{2\iota _2 \text {sec}^2\left( \sqrt{-\iota _2} (x-\nu t)\right) }{\frac{\varkappa }{12 \eta _2}-\left( \frac{\varkappa }{24 \eta _2}-\frac{\varkappa }{24 \eta _2}\right) \text {Sec}^2\left( \sqrt{-\iota _2} (x-\nu t)\right) }}\right) e^{i \left( -\beta x+\omega t-\varrho ^2 t + \varrho W(t) \right) },\nonumber \\ & \iota _2<0,\hspace{0.2cm}\iota _6>0,\hspace{0.2cm}\varkappa >0. \end{aligned}$$

where $$\varkappa =\sqrt{3} \sqrt{a_1^4 \left( 3 \eta _5^2-8 \eta _2 \eta _4\right) }-3 a_1^2 \eta _5$$.

For bright solitons, Eq. (32) offers a stochastic solution, whereas Eq. (33) gives a singular periodic stochastic solution.

## White noise influence on some retrieved solitons

**Fig. 1:**
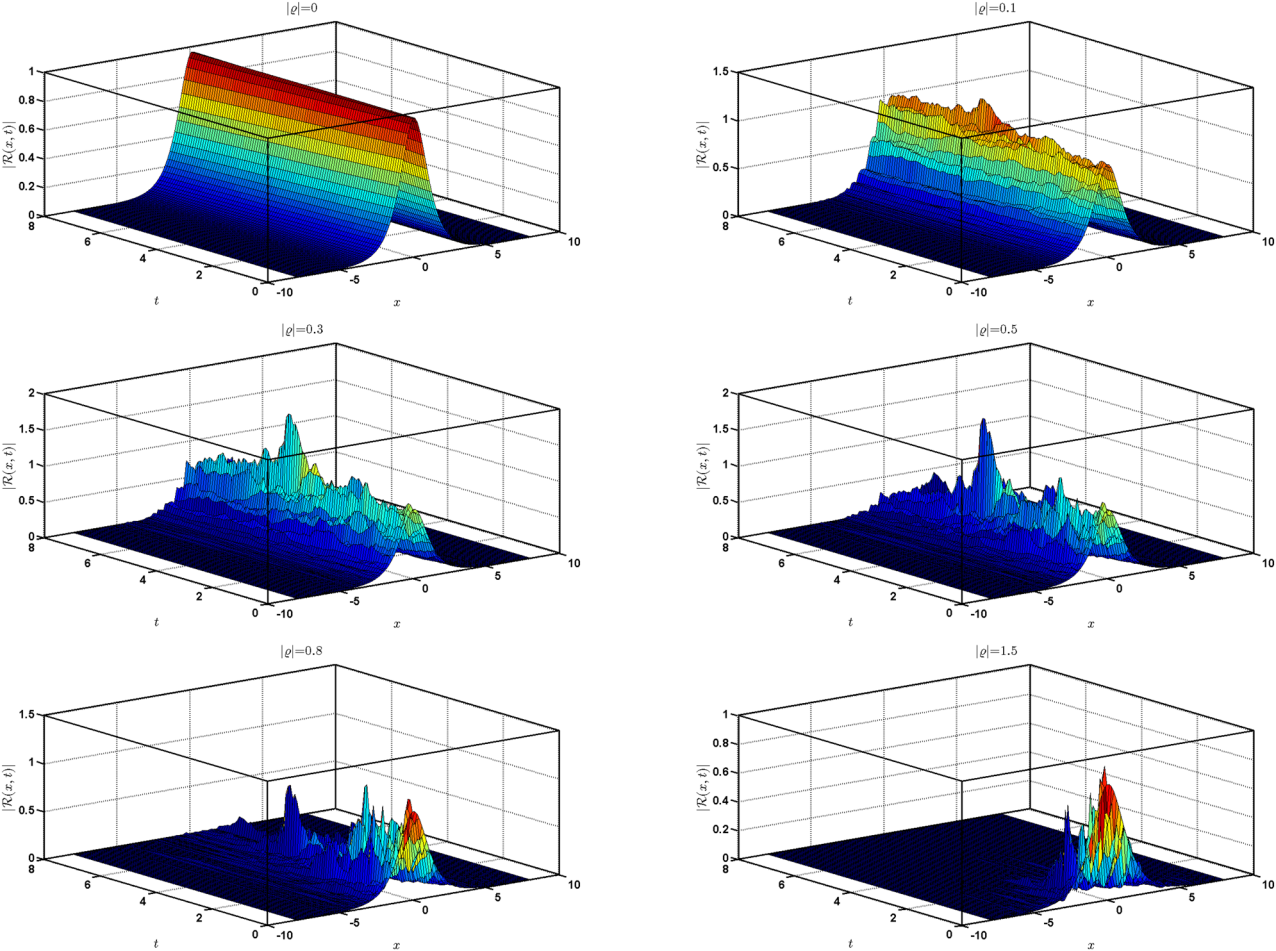
3D graphical depictions of Eq. (13) with $$\eta _1=1.84,$$$$~\eta _2=0.29,$$$$~\eta _4=\eta _5=2,$$$$~\eta _6=1.27,$$$$~\omega =-0.64,$$$$~\iota _4=-2$$. The noise intensity values corresponding to the six images are, in order: $$\varrho = 0, 0.1, 0.3, 0.5, 0.8, 1.5$$.

**Fig. 2:**
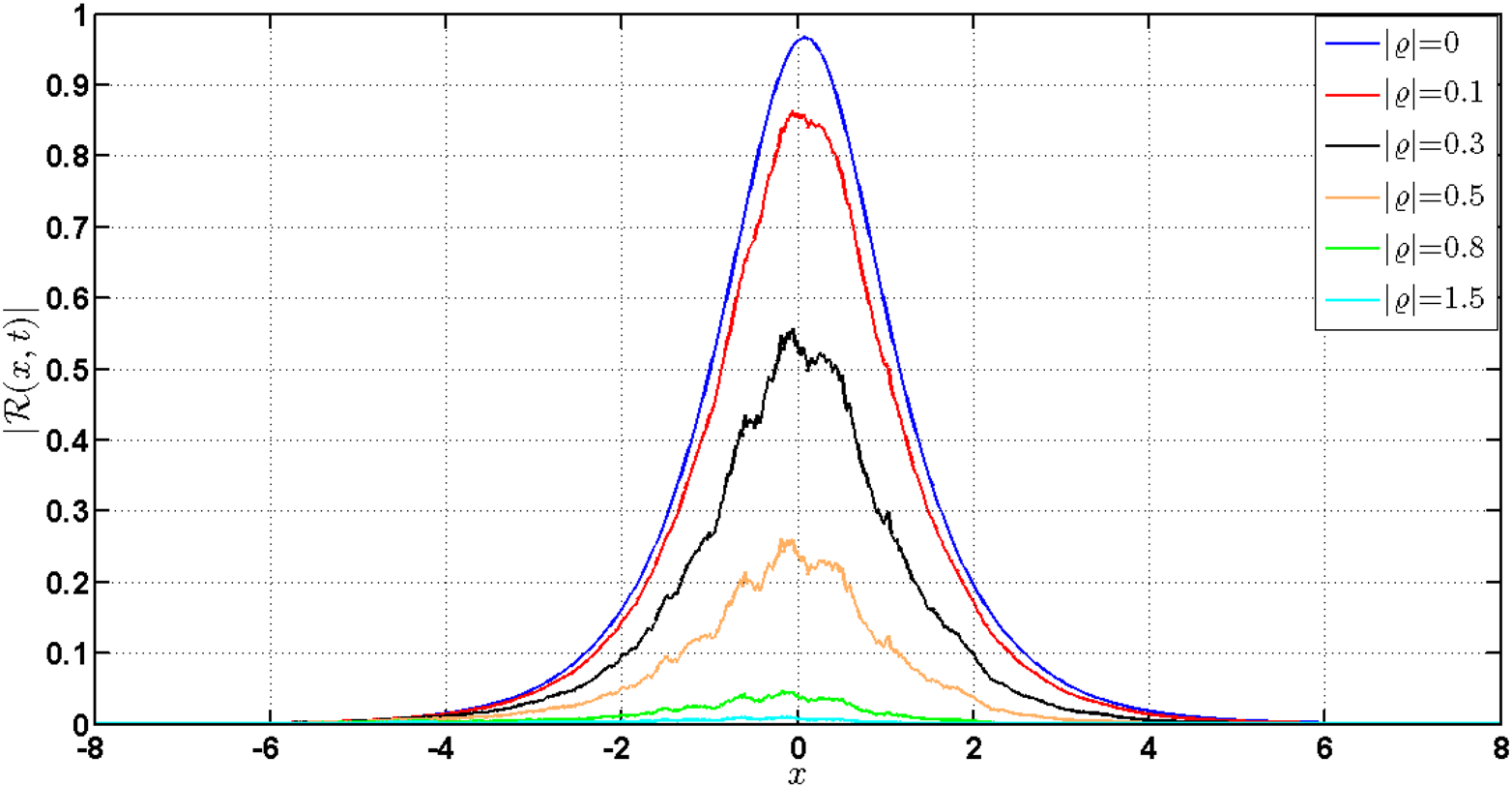
2D graphical depictions of Eq. (13) with $$\eta _1=1.84,$$$$~\eta _2=0.29,$$$$~\eta _4=\eta _5=2,$$$$~\eta _6=1.27,$$$$~\omega =-0.64,$$$$~\iota _4=-2$$.

**Fig. 3:**
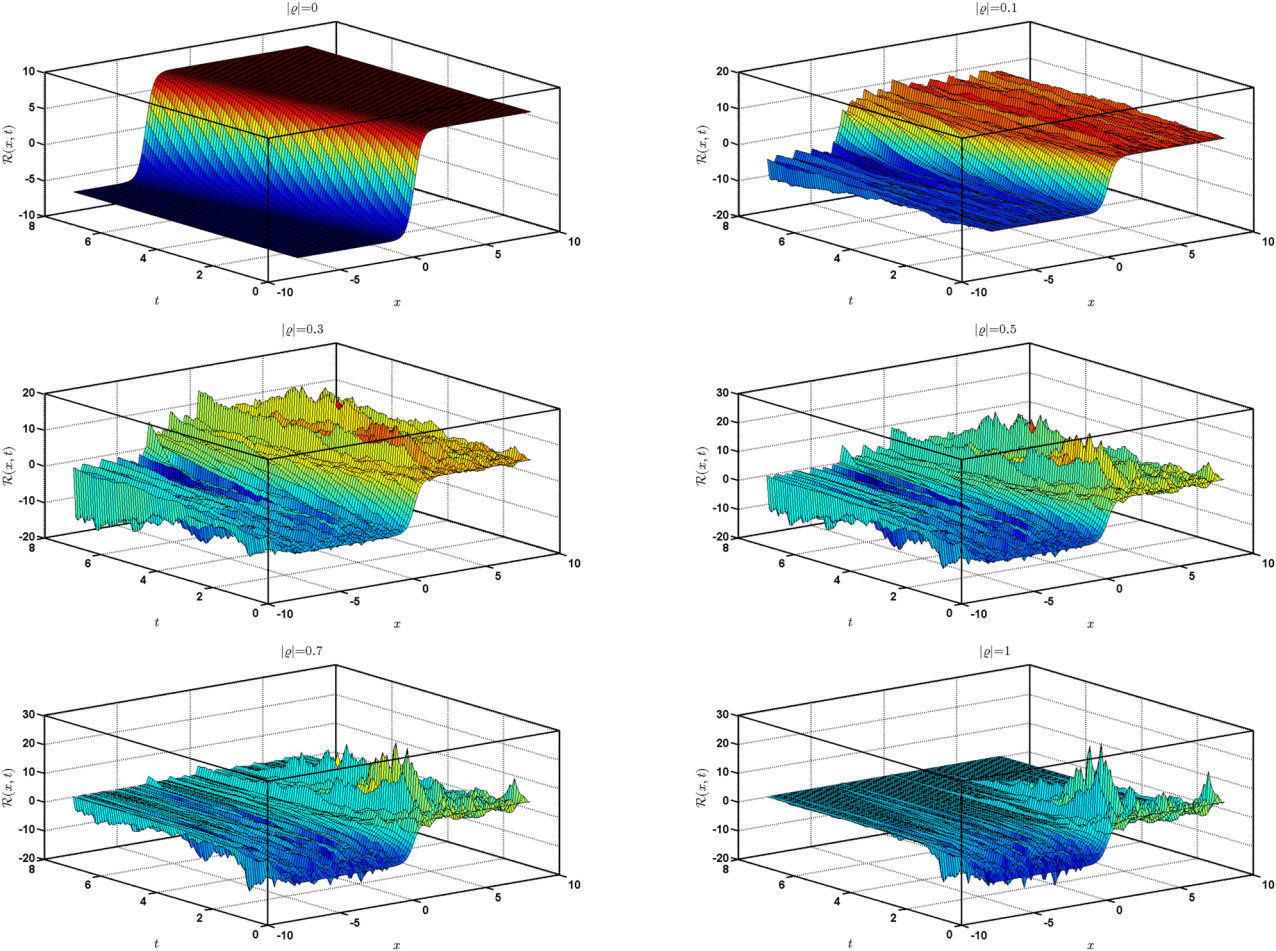
3D graphical depictions of Eq. (19) with $$\eta _1=-0.45,$$$$~\eta _2=0.08,$$$$~\eta _4=-0.17,$$$$~\eta _5=-0.38,$$$$~\eta _6=-0.49,$$$$~\omega =0.86,$$$$~\iota _4=1.1$$. The noise intensity values corresponding to the six images are, in order: $$\varrho = 0, 0.1, 0.3, 0.5, 0.7, 1$$.

**Fig. 4:**
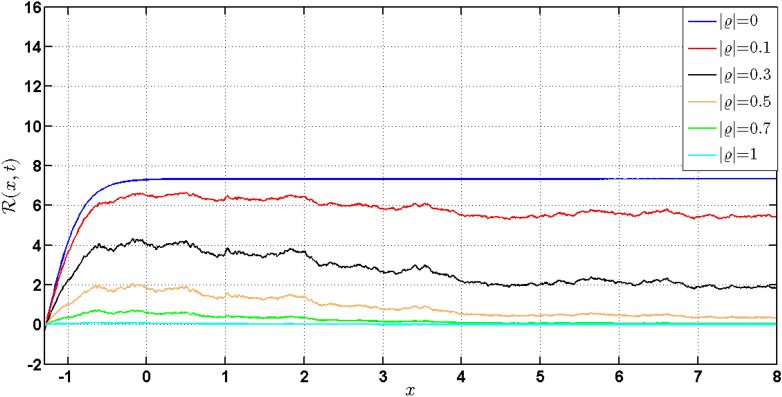
2D graphical depictions of Eq. (19) with $$\eta _1=-0.45,$$$$~\eta _2=0.08,$$$$~\eta _4=-0.17,$$$$~\eta _5=-0.38,$$$$~\eta _6=-0.49,$$$$~\omega =0.86,$$$$~\iota _4=1.1$$.

This section investigates the influence of additive white Gaussian noise on the obtained analytical solutions. The numerical simulations are carried out using MATLAB (R202x), where the exact closed-form solutions are evaluated on a discretized spatial–temporal grid using a uniform mesh. A sufficiently fine grid is employed to ensure numerical stability and accuracy of the graphical representations.

To incorporate stochastic perturbations, additive white Gaussian noise of the form

$$\mathcal {R}_{\text {noisy}} = \mathcal {R}(x,t) + \varrho \, \mathcal {N}(0,1)$$

is introduced, where $\varrho$ denotes the noise intensity and $\mathcal {N}(0,1)$ is a standard normal random variable generated using MATLAB’s built-in function randn. The simulations are repeated for different values of $\varrho$ to examine the robustness and stability of the derived wave structures under perturbations.

The analytical expressions are directly evaluated without approximation errors from numerical solvers, while noise is superimposed explicitly at each grid point. The resulting data are visualized using MATLAB’s plotting tools to generate both 2D and 3D surface plots.

For validation purposes, the obtained numerical profiles are first checked against the deterministic case ($\varrho = 0$), where the results recover the exact analytical wave structures reported in the previous sections. In addition, the qualitative behavior of the solutions–such as amplitude decay and surface flattening under increasing noise–is consistent with previously reported results in similar nonlinear wave models and perturbation analyses in the literature, confirming the physical reliability of the obtained solutions.

For Eq. (13), the parameter set $\eta _1=1.84,~\eta _2=0.29,~\eta _4=\eta _5=2,~\eta _6=1.27,~\omega =-0.64,~\iota _4=-2$ is used to generate the 3D profiles shown in Fig. (1), while the corresponding 2D profiles are shown in Fig. (2). Similarly, for Eq. (19), the parameter set $\eta _1=-0.45,~\eta _2=0.08,~\eta _4=-0.17,~\eta _5=-0.38,~\eta _6=-0.49,~\omega =0.86,~\iota _4=1.1$ is used to generate Figs. (3) and (4). In both cases, increasing the noise intensity leads to a gradual reduction in peak amplitude and smoothing of the wave structures, indicating the expected damping effect of stochastic perturbations.

## Linear stability analysis

Linear stability analysis is a powerful technique for investigating the response of nonlinear wave solutions to small perturbations^37^. It provides a systematic framework to determine whether a given steady-state (plane-wave) solution persists under weak disturbances or evolves into an unstable regime. In particular, the temporal behavior of infinitesimal perturbations offers direct insight into the modulational properties and physical robustness of the obtained solution.

To study this behavior, we perturb the constant background solution $R_0$ by introducing a small disturbance of the form

34 $$\begin{aligned} \mathcal {R} = R_0 + \epsilon \phi (x,t), \qquad 0 < \epsilon \ll 1, \end{aligned}$$

where $\phi (x,t)$ represents a complex-valued perturbation and $\epsilon$ is a small bookkeeping parameter.

Substituting this expansion into Eq. (1), expanding all nonlinear terms, and retaining only terms up to first order in $\epsilon$, we obtain the following linearized evolution equation for the perturbation:

35 $$\begin{aligned} \begin{aligned}&R_0^2 \eta _3 \phi ^*_x + 2 R_0^4 \eta _4 \phi ^*_x + 2 R_0^2 \eta _3 \phi + 3 R_0^4 \eta _4 \phi + i \phi _t \\&- i \eta _6 \phi _x - i (2\eta _7 + \eta _8) R_0^2 \phi _x + R_0^2 \eta _5 \phi _{xx} + i \eta _1 \phi _{xxx} + \eta _2 \phi _{xxxx} = 0. \end{aligned} \end{aligned}$$

We assume a normal mode perturbation of the form

36 $$\begin{aligned} \phi (x,t) = a e^{i(kx - \omega t)}, \qquad \phi ^*(x,t) = b e^{-i(kx - \omega t)}, \end{aligned}$$

where *k* is the wave number and $\omega$ is the perturbation frequency.

Substituting into the linearized equation and separating the modes $e^{\pm i(kx-\omega t)}$, we obtain a consistency condition for the amplitudes. From the conjugate mode, we obtain the constraint

37 $$\begin{aligned} b R_0^2 \eta _3 + 2 b R_0^4 \eta _4 = 0. \end{aligned}$$

Finally, the remaining terms yield the dispersion relation governing the evolution of perturbations:

38 $$\begin{aligned} \omega = R_0^4 \eta _4 - k \Big ( k^2 \eta _1 + k^3 \eta _2 - k R_0^2 \eta _5 + \eta _6 + R_0^2 (2\eta _7 + \eta _8) \Big ). \end{aligned}$$

**Fig. 5:**
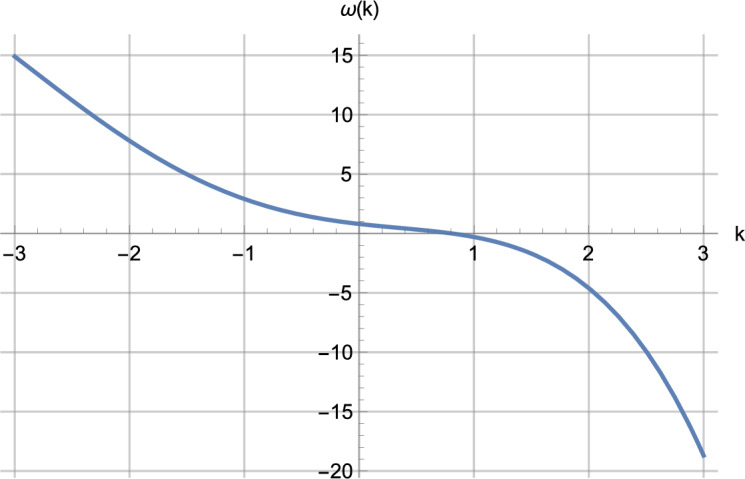
Dispersion relation $\omega (k)$ showing the dependence of frequency on the wave number *k*.

To further illustrate the dispersive characteristics of the system, the dispersion relation $\omega (k)$ is plotted in Fig. (5) using the parameter set $R_0=1$, $\eta _1=0.5$, $\eta _2=0.1$, $\eta _4=0.8$, $\eta _5=0.6$, $\eta _6=0.3$, $\eta _7=0.2$, and $\eta _8=0.4$. The obtained dispersion curve is smooth and purely real for real values of *k*, indicating the absence of any exponential growth or decay of perturbations.

Therefore, the perturbations remain purely oscillatory in time, and the system does not exhibit modulational instability at the linear level. This implies that the constant background solution is linearly neutrally stable, and disturbances propagate as dispersive waves governed by the real dispersion relation.

## Conclusion

This work aimed to investigate a high-order SNLSE in a non-Kerr law medium with weak non-local nonlinearity. This goal was formalized by taking into consideration the MEMM and a wave variable transformation to accurately extract the model’s optical solitons. The study’s outcomes showcased the exceptional capability of the MEMM in addressing high-order nonlinear Schrödinger problems. The developed approach offers a wide variety of stochastic solutions, including periodic, singular periodic, hyperbolic, rational, bright, dark, and singular solitons. The 2D and 3D graphs were used to depict the physical characteristics of different solution types of a few chosen solutions.

When compared with deterministic results (i.e., solutions without the stochastic term), the stochastic solutions reveal significant modifications in soliton amplitude, width, and stability due to noise intensity, highlighting the impact of stochastic perturbations on the optical solitons.

Although MEMM provides a versatile framework for deriving explicit solutions, it has some limitations: it may not capture all solution branches in strongly nonlinear or highly stochastic regimes, and careful parameter selection is required to ensure physical relevance, and the method can become increasingly complicated for higher balances (i.e., larger values of *N* in the solution ansatz), making analytical computations more challenging.

When compared with previous research findings, the obtained results demonstrate the efficiency, originality, and accuracy of the proposed method for a variety of nonlinear partial differential equations arising in mathematical physics.

## Data Availability

The datasets used and/or analyzed during the current study are available from the corresponding author upon reasonable request.
